# Historical sampling reveals dramatic demographic changes in western gorilla populations

**DOI:** 10.1186/1471-2148-11-85

**Published:** 2011-04-01

**Authors:** Olaf Thalmann, Daniel Wegmann, Marie Spitzner, Mimi Arandjelovic, Katerina Guschanski, Christoph Leuenberger, Richard A Bergl, Linda Vigilant

**Affiliations:** 1 Max Planck Institute for Evolutionary Anthropology, Deutscher Platz 6, 04103 Leipzig, Germany; 2 Dept. of Ecology and Evolutionary Biology, University of California Los Angeles, 621 Charles E. Young Dr South, Los Angeles, CA 90095, USA; 3 Département de mathématiques, Université de Fribourg, 1700 Fribourg, Switzerland; 4 North Carolina Zoological Park, 4401 Zoo Parkway, Asheboro, NC 27205, USA; 5 Division of Genetics and Physiology, Department of Biology, University of Turku, Vesilinnantie 5, 20014 Turku, Finland

## Abstract

**Background:**

Today many large mammals live in small, fragmented populations, but it is often unclear whether this subdivision is the result of long-term or recent events. Demographic modeling using genetic data can estimate changes in long-term population sizes while temporal sampling provides a way to compare genetic variation present today with that sampled in the past. In order to better understand the dynamics associated with the divergences of great ape populations, these analytical approaches were applied to western gorillas (*Gorilla gorilla*) and in particular to the isolated and Critically Endangered Cross River gorilla subspecies (*G. g. diehli*).

**Results:**

We used microsatellite genotypes from museum specimens and contemporary samples of Cross River gorillas to infer both the long-term and recent population history. We find that Cross River gorillas diverged from the ancestral western gorilla population ~17,800 years ago (95% HDI: 760, 63,245 years). However, gene flow ceased only ~420 years ago (95% HDI: 200, 16,256 years), followed by a bottleneck beginning ~320 years ago (95% HDI: 200, 2,825 years) that caused a 60-fold decrease in the effective population size of Cross River gorillas. Direct comparison of heterozygosity estimates from museum and contemporary samples suggests a loss of genetic variation over the last 100 years.

**Conclusions:**

The composite history of western gorillas could plausibly be explained by climatic oscillations inducing environmental changes in western equatorial Africa that would have allowed gorilla populations to expand over time but ultimately isolate the Cross River gorillas, which thereafter exhibited a dramatic population size reduction. The recent decrease in the Cross River population is accordingly most likely attributable to increasing anthropogenic pressure over the last several hundred years. Isolation of diverging populations with prolonged concomitant gene flow, but not secondary admixture, appears to be a typical characteristic of the population histories of African great apes, including gorillas, chimpanzees and bonobos.

## Background

How species form and change over time is one of the central preoccupations of evolutionary biology (e.g [1,2]). Many species exist today in distinct populations whose size and interconnectivity have changed over time, in response to both recent and ancient climate changes and human activities. New advances in the analysis of patterns of genetic variation found in contemporary populations allow the disentanglement of the combined effects of these forces to reveal the recent and long-term histories of those populations. Population histories, including the timing of population divergences, the direction and extent of migration and estimates of effective population sizes, can be effectively inferred using explicit modeling approaches based upon coalescent theory (e.g. [3]).

These approaches combine information from neutrally-evolving loci sampled across the genome and employ models that can allow for a variety of population divergence scenarios. The classic allopatric model of speciation suggests that new species form when populations become isolated from one another [4]. However, subdivision of species or populations may not happen abruptly or completely, and divergence can occur even while some degree of gene flow may continue. Models of isolation that incorporate migration must, therefore, also be tested [5]. In addition, gene flow, even among evolutionarily divergent lineages, is routine at the contact zones of many taxa including lizards, ungulates and primates (e.g., baboons [6,7], macaques [8]), with convincing evidence even suggesting that some species arose from hybridization of two different species [9,10]. Hence, other speciation models also allow for the possibility of resumption of gene flow following population divergence.

In order to gain an appreciation for the tempo, mode, timings and ultimate causes of species and population divergences, it is advisable to first focus on representatives of a group of closely related species in order to uncover general trends. As our closest living relatives, the African great apes are particularly fascinating and have been well-studied in the past. For example, the closely-related chimpanzee (*Pan troglodytes*) and bonobo (*Pan paniscus) *species are suggested to have diverged some 1.5 million years ago (mya), and, not surprisingly given their long-term separation by the formidable barrier of the Congo River, there are no signs of recent gene flow between the species, although ancient gene flow was likely [11,12]. The three regional populations or subspecies of chimpanzees diverged on the order of half a million years ago, but the eastern and central populations exchange migrants at appreciable rates, while recent unidirectional migration from western to central chimpanzees is also inferred [12,13]. The central chimpanzee (*Pan troglodytes troglodytes*) population is suggested to be the ancestral population of all chimpanzees based on the findings of its larger effective population size and signals of population bottlenecks associated with the divergence of the other subspecies [12]. Overall, the *Pan *species and chimpanzee subspecies appear to have evolved following processes of isolation, limited migration, and population expansions.

**Figure 1:**
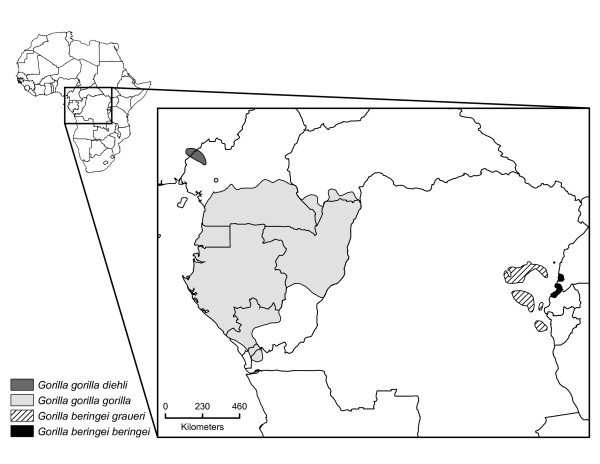
**Gorilla distribution map**. Approximate current distribution of gorillas in Equatorial Africa. *Gorilla gorilla gorilla *refers to western lowland gorillas and *Gorilla gorilla diehli *to Cross River gorillas. East African gorillas are divided into *Gorilla beringei beringei*, also known as mountain gorillas and *Gorilla beringei graueri*, known as eastern lowland gorillas.

Like chimpanzees, gorillas occur in central equatorial Africa but are somewhat less widespread. The contemporary distribution of gorillas features a pronounced gap of several hundred kilometers between western gorillas (*Gorilla gorilla*) and eastern gorillas (*G. beringei*) (Figure 1), yet these two species retain behavioral and morphological similarities and were until recently considered subspecies of the same species [14,15]. In previous work, we inferred from genetic data that the initial population divergence of western and eastern gorillas might have occurred approximately 0.9-1.6 mya [16], but that gene flow in both directions had persisted until as recently as 78,000 years ago [17]. Whereas these studies contributed to our understanding of the evolutionary history of gorillas on an interspecific level, little is known about population size changes in the past and the intraspecific evolutionary dynamics of gorilla populations. Intriguingly, a recent survey of skull and dental morphology of museum specimens suggested that some observed craniofacial anomalies may represent the outcome of secondary admixture between separate populations in both western and eastern gorillas, but this scenario has not yet been explicitly tested with genetic data [18]. While analyses of genetic data from great apes tend to favor population histories featuring isolation with migration rather than secondary introgression, such admixture is suspected to play a role in the evolution of various old world monkey taxa [19] as well as in the recent history of modern humans and Neanderthals [20].

The focus of this study is a comprehensive assessment of the evolutionary histories of the broadly distributed population of western lowland gorillas (*G. g. gorilla*) and the range restricted, Critically Endangered Cross River gorillas (*G. g. diehli*). The current census population size of western lowland gorillas is on the order of tens of thousands (IUCN Red List of Threatened Species™). In contrast, Cross River gorillas consist of approximately 200-300 individuals in fragmented populations in a highland region on the Nigeria-Cameroon border [21]. Previous genetic analyses of Cross River gorillas using multiple autosomal microsatellite loci applied to DNA derived from non-invasively collected fecal samples suggested a recent and/or severe population decrease [22]. Here we build upon this work by using DNA obtained from museum specimens to directly compare the genetic diversity observed today with that existing approximately 100 years ago. We next employ a highly efficient coalescent-based approach that takes advantage of both our temporal sampling scheme and use of multiple independently-evolving loci. This allows us to reconstruct the evolutionary relationship of Cross River and western lowland gorillas by estimating the time of their divergence, the occurrence and duration of gene flow between them, and their current and ancestral effective population sizes. To evaluate different evolutionary scenarios we specifically test two alternative models, one of which allows for divergence in the presence of gene flow [23] while the other encompasses a secondary admixture scenario by permitting divergence in isolation and subsequent resumption of genetic exchange or admixture between the two populations [18].

## Results and Discussion

### Recent changes in genetic diversity

In order to assess the patterns of genetic diversity within and between two temporally spaced Cross River gorilla population samples, we generated microsatellite genotypes for 14 approximately 100-year-old Cross River museum specimens (Additional file 1: Table S1) and compared these data with published results from 71 non-invasively collected contemporary Cross River gorillas [24].

The low concentration and fragmented nature of DNA extracted from sources such as fecal samples or historical remains can lead to inaccurate microsatellite genotyping through the stochastic nonamplification of an allele at a heterozygous locus ('allelic dropout') or consistent nonamplification of an allele due to mutations at a priming site ('null alleles') [25,26]. Such errors can potentially bias conclusions concerning a population's genetic diversity or demographic history. We found no evidence of problems with any of the loci amplified in the historical samples, while two loci in the published modern Cross River data set potentially contained null alleles. This suggestion of null alleles disappeared when sampling localities were considered and the pattern was attributed to the existence of genetic structure in contemporary Cross River gorillas [24]. The analyses presented here provide further evidence for this conclusion. For instance, patterns of Hardy-Weinberg equilibrium (HWE) and linkage disequilibrium differed between the two data sets. Specifically, three loci from the modern data set (D5S1470, D7S817, D8S1106) deviated significantly from HWE in a locus-wise probability test, a test that was also highly significant when performed across all loci (corrected p < 0.001). Furthermore, the modern Cross River samples approached a significant 'deficit of heterozygotes' when using a global test over all loci (corrected p = 0.071). Significant linkage disequilibrium was observed for 14 of the 28 locus by locus comparisons in the modern data set (corrected p < 0.0042), but for none of the loci in the historical data, when corrected for multiple testing. In addition, although not statistically significant, the mean observed heterozygosity (H_o_) was higher in the historical dataset than the modern one (0.732 and 0.631, respectively) and F_IS _values increased from -0.05 in the historical to a significant 0.072 in the contemporary sample (SI: Tables S2 and S3). In summary, these results are most readily explained by a Wahlund effect due to pooling of the samples from the structured, contemporary population [27].

The average expected heterozygosity (H_e_) was 0.701 and 0.679 for the historical and contemporary samples, respectively (SI: Table S2). Because the sample sizes differed, we resampled 14 genotypes from the contemporary samples 10,000 times and estimated mean H_e_. We obtained a higher or equal mean H_e _than observed in the historical sample in only 10.6% of the cases. Consistent with this result we observed a trend towards higher allelic richness in the historical samples compared to the modern samples in five of eight loci (SI: Table S3). A test for population differentiation assuming the same distribution of alleles within the two populations approached significance (p = 0.063), and is in agreement with the low F_ST _value (F_ST _= 0.0071, p = 0.199). In sum, the lack of statistical significance of the comparisons of the standard summary statistics may reflect low power due to the limited number of samples and loci, while the tendency towards higher variation in the past may reflect genetic drift or a population size decrease in the Cross River gorillas over the last 100 years, a finding that has been observed in other large mammals such as whales and orangutans and is usually attributed to increasing anthropogenic pressure [28,29].

### Temporal estimates of effective population sizes

Estimates of a population's effective size (N_e_) - the size of an idealized Wright-Fisher population [30,31] resulting in the same genetic variability as observed in the population examined [32] - highlight the strength of genetic drift acting on the genetic variation and is considered in assessments of a population's viability [33]. We used multiple analytical approaches to estimate the effective population sizes of the Cross River gorillas. First we used short-term estimators based on the changes in allele frequencies over the last hundred years. Two such moment estimators of N_e _yielded values of 221 and 276, while a pseudo-likelihood estimator produced a similar value of 244 (Table 1).

**Table 1:** Estimates of the short-term effective population size (N_e_) of the Cross River gorilla population

	Method	N_e _point estimator	95% boundaries
	Moment estimators	**221**^1^	n.d.
		**276**^2^	57 - infinity
	Pseudo-likelihood	**244**^3^	86 - infinity
	Coalescence	**1,626**^4^	165 - infinity
		**1,142**^5^	193 - 2,792

^1 ^calculated with ***MNE***; no 95% limits provided (n.d.)

^2 ^ref. [63]

^3 ^ref. [61]

^4 ^refs. [34,64]

^5 ^ref. [34]

As complex models use more information, it has been suggested that likelihood- and coalescence-based methods may outperform moment estimators of effective population sizes in precision and accuracy [34,35]. Our N_e _estimates derived from two probabilistic approaches yielded substantially higher values of 1,626 and 1,142 (Table 1). However, complex coalescence-based models are highly dependent on underlying assumptions. The apparent discrepancy between the various N_e _estimators might be aggravated by the complexity of gorillas' life history and demography and potentially reflect deviations from a standard Wright-Fischer model. One of the most unrealistic assumptions is that of panmixia in the focal population, a situation rarely applicable to social animals. Second, the assumption of discrete generations is often violated due to the existence of age-structured, social groups containing several overlapping generations. This is especially the case in great apes, where closely related individuals (parent-offspring pairs) are found together in one group and dispersal distances are likely limited [36,37]. In sum, our results are consistent with the varied sensitivities of N_e _estimators and confirm the utility of comparing results from multiple estimators [38] or using modeling approaches to infer demographic histories.

### Demographic simulations and gorilla population histories

**Figure 2:**
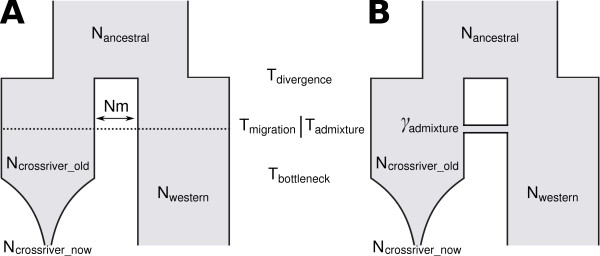
**Schemes of the demographic models**. Schemes of the models describing the evolutionary history of western gorillas. A) Divergence accompanied with gene flow model; B) Divergence and secondary admixture model. For a detailed description see text and Supplementary Information.

In order to investigate the deeper demographic history of Cross River gorillas we compared two potential demographic scenarios: a divergence with secondary admixture model and a model of isolation with migration (Figure 2). Both models allowed for a severe, recent population decline in the Cross River population and incorporated contemporary and museum samples appropriately.

**Figure 3:**
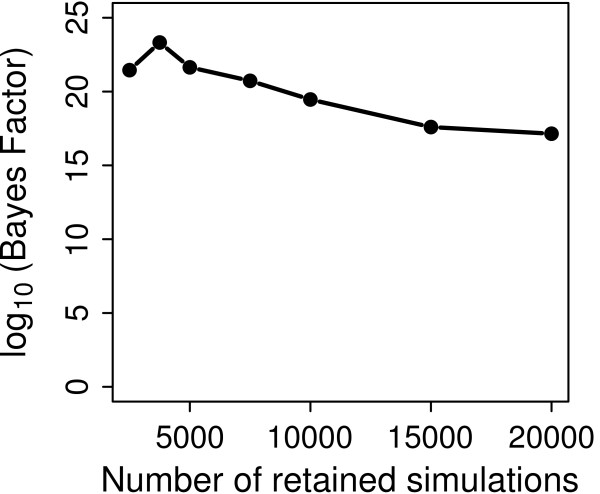
**Bayes factor robustness**. Given are estimates of the Bayes factor on the log_10 _scale when comparing the migration model to the secondary admixture model (see text for further details), according to the number of retained simulations.

It has recently been suggested that the existence of genetic structure in a population might generate "spurious" bottleneck signals in demographic simulations [39-41]. This erroneous inference can be understood by considering the coalescent process in a subdivided population. As shown by [42], the coalescence process may be divided into two distinct phases: During the "scattering phase", lineages either coalesce or migrate quickly to demes other than those being sampled, until there is a single lineage left per deme. In the "collecting phase", the remaining lineages follow a standard coalescence process, like in a single population, but on a different time scale. If many samples are drawn from the same deme, the rate of coalescent events in the scattering phase is large, resulting in a genealogy that will look much like that expected after a recent bottleneck [5,39]. Since we found limited population structure in the contemporary sample, only one haplotype per sampled individual was considered in all our demographic simulations. This effectively reduces the number of alleles sampled from the same deme and thus reduces the potential bias in our inference. Despite the resulting reduced sample size we could safely reject the secondary admixture model in favor of the model of isolation with migration (log10Bayes factor ~ 22) (Figure 3).

We next estimated parameters of the preferred isolation with migration model, including the timing of divergence between the Cross River and western lowland gorillas, timing of cessation of gene flow between these populations, onset of population size changes and the contemporary and ancestral effective population sizes (Figure 2A).

**Figure 4:**
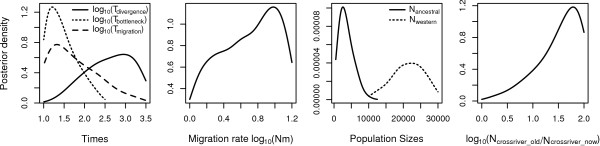
**Posterior distribution of demographic parameters**. Posterior distribution of demographic parameters as estimated in the divergence with gene flow model including migration between Cross River and western lowland gorillas (log_10_[N_m_]), ratio of ancestral to current effective size of Cross River gorillas (log_10_[N_CR OLD_/N_CR NOW_], effective population sizes and times of various demographic events, respectively. Times are given in log_10_-scale of generations and population sizes refer to the effective size of the respective population.

The effective size of the ancestral population of Cross River and western lowland gorillas was estimated at 2,547 (95% HDI: 500, 7,684; Figure 4 and Table 2). This ancestral population may have diverged into Cross River and western lowland gorillas at least 890 generations or 17,800 years ago (95% HDI: 760, 63,245 years). The effective population size of the western lowland gorillas subsequently increased approximately 9-fold to an estimated current day 22,376 (95% HDI: 12,879, 29,532). The effective size of the ancestral Cross River gorilla population was also large initially, probably caused by an expansion in space and numbers but beginning at about 320 years ago (95% HDI: 200, 2,825 years) it decreased by approximately 60-fold to 271 (95% HDI: 122, 300). We observed a signature of substantial gene flow between western and Cross River gorillas that continued after the initial divergence (2N_m _≈ 9.5 or roughly 4.5 individuals per generation) but ceased at around 21 generations or 420 years ago (95% HDI: 200, 16,256 years), at approximately the same time as the inferred onset of the population bottleneck.

**Table 2:** Estimates of parameters of the demographic model applied to Cross River and western lowland gorillas

	Prior distribution	min	max	Posterior mode	HDI 50^b^	HDI 90^b^	HDI 95^b^
2Nm	Loguniform	1	15.85	9.55	[4.57, 13.8]	[1.58, 15.84]	[1.32, 15.85]
N_crossriver_old_/N_crossriver_now_)	Loguniform	1	100	61.7	[33.1, 93.3]	[10, 100]	[4.2, 100]
N_crossriver_now_	N(200, 100) ^a^	68	300	271	[223, 292]	[146, 300]	[122, 300]
N_ancestral_	Uniform	500	25,000	2,547	[1,383, 4,032]	[500, 6,681]	[500, 7,684]
N_western_	N(24,000, 5000) ^a^	10,000	30,000	22,376	[18,765, 25,319]	[14,217, 28,930]	[12,879, 29,532]
T_divergence_	Loguniform	10	3,162	891	[269, 1,738]	[60.3, 3,090]	[38, 3.162]
T_bottleneck_	Loguniform	10	316	16	[33.1, 93.3]	[10, 97.7]	[10, 141]
T_migration_	Loguniform	10	3,162	21	[11.7, 60.3]	[10, 446.7]	[10, 812]

N_crossriver_now_, N_ancestral_, N_western _represent the effective population sizes of Cross River, ancestral and western lowland gorillas respectively. Timings in generations were estimated on the log_10 _scale and indicate the divergence (T_divergence_), the onset of the bottleneck (T_bottlneck_) and the cessation of migration (T_migration_). The number of diploid individuals exchanged between the populations was also estimated on the log_10 _scale as 2 Nm. For parameters estimated on the log_10 _scale we chose uniform priors on the same scale.

^a ^Corresponds to a normal distribution of the form N(μ, σ) truncated at [min, max]

^b ^The high posterior density interval HDI is chosen as the smallest continuous interval spanning 50% of the posterior surface. The other HDI are chosen accordingly.

The large credibility intervals on our posterior parameters suggest that further investigations using more loci or individuals may be useful to refine the demographic model. However, we rigorously validated our inferences and found only slight biases for some of the estimates (see SI: Supplementary Figure.3): an overestimation of the current Cross River population size, an underestimation of the divergence time and a more global overestimation of the ancestral gorilla population size over the entire parameter space. These minor biases do not substantially influence our inferences, which are associated with large posterior density intervals but nonetheless reveal a strong recent decrease in the effective size of the Cross River population.

The current analysis contributes to a composite view of gorilla population history based on genetic data (Table 3). An initial split of western and eastern gorillas about one million years ago was accompanied by bidirectional gene flow until as recently as 78,000 years ago [17]. Some 17,800 (95% HDI 760, 63,245) years ago, western lowland and Cross River gorillas diverged but substantial gene flow between the two western gorilla subspecies ceased only about 420 (95% HDI 200, 16,256) years ago. A marked decline of Cross River gorilla population size began only about a hundred years later, resulting in a current estimated effective population size of 271 (95% HDI 122-300). Such a pronounced, recent reduction in size accounts for the high concordance of effective population and census size as it drastically reduces variation and prolongs gene coalescences. These demographic events, along with the signals of likely population structure in the ancestral western gorilla population [17], are consistent with a scenario of changing climate conditions over the last tens of thousands of years that led to repeated expansion and contraction of forests, and hence of forest-dwelling ape populations, as well as more recent increased anthropogenic impact.

**Table 3:** Estimates of effective population sizes for the indicated gorilla populations

Population	Method	N_e _estimates
Cross River^1^	temporal	~250
Cross River^1^	ABC	271
Western lowland^1^	ABC	22,376
Western gorillas^2*^	IM	17,700
Western gorillas^2*^	θ_W_	24,100
Western gorillas^3*^	MIMAR	13,000
Western lowland and Cross River (ancestral) ^1^	ABC	2,547

^1 ^This work

^2 ^ref. [17]

^3 ^ref. [13]

* These studies included a single Cross River gorilla in their dataset

Africa's climate during the late Pleistocene was characterized by oscillations of aridity and humidity leading to a continuous cycle of forest expansion and contraction and the creation of forest refugia during arid phases [43]. Patterns of genetic diversity in gorillas and other mammals have been suggested to reflect the effects of refugial fragmentation during the last glacial maximum [44,45], and one such forest refugia has been suggested to have existed in the Cross River area. Consequently, the isolation of this region from other forest refugia in western equatorial Africa might have promoted the divergence of Cross River gorillas from their ancestral population ~17,800 years ago. Although the mode estimate of the eventual cessation of gene flow between western lowland and Cross River gorillas is rather recent, its 95% HPD interval includes major climatic changes in the region around 3,000-2,500 years ago that led to increased aridity and the expansive replacement of forests by grasslands [46,47].

In addition to climate change, human activities have likely had an increasing impact upon the Cross River gorillas in recent times. Initial settlement of the northwestern region of Cameroon by Bantu agriculturalists has been estimated to have coincided with contraction of forests in the region and occurred as early as 2,500 years ago [47]. Aside from habitat destruction, hunting is another anthropogenic threat that has likely caused the apparent population size decrease of Cross River gorillas over the last centuries. Firearms were introduced to the region beginning in the 18^th ^century and increased in both numbers and sophistication starting in the mid to late 19^th ^century [48]. Additionally, beginning in the late 19^th ^century, the region underwent a rapid growth in human population density, increasing tenfold between 1900 and 2000 [49]. These two factors together almost certainly created a significant intensification of hunting pressure on large mammals like gorillas [21,22]. Notably, the inferred population size decrease in the history of Cross River gorillas by a factor of ~60 translates into a loss of ~23% of the gorilla population per generation since the onset of the bottleneck some 16 generations ago. Although this is a substantial decrease in size, other equatorial African gorilla populations have been described to suffer even more dramatic size reductions. In 2003, Walsh and colleagues reported a 56% decline in the population size of gorillas in Gabon over the last 20 years (or approximately a single generation) [50]. The authors concluded that the cause of this severe population size crash is a synergism between human activities, including hunting, habitat loss due to deforestation and disease (see also [51]) and strongly emphasized the need for effective and immediate conservation actions in order to ensure the long-term survival of gorilla populations.

Hunting of gorillas continues today and represents one of the greatest threats to their persistence [21]. Particularly for small populations that may have gone through recent reductions in size, effective enforcement of anti-poaching laws and maintenance of existing habitat to allow the population to stabilize and expand is crucial to their future survival [21]. While such measures will facilitate the short-term survival of Cross River gorillas, the impact of reduced levels of genetic diversity on their long-term viability remains unclear. The weak signal for loss of genetic variation in the Cross River gorilla population is consistent with the recent population bottleneck we identified, as the number of generations elapsed may not yet have been sufficient to significantly reduce levels of heterozygosity or allelic richness. The relatively short duration but dramatic severity of the bottleneck also explains the concordance between estimated census population size and our estimates of N_e_, which often differ by up to a factor of ten in wildlife populations [31]. Given this relationship between N and N_e_, it is possible that if the population were allowed to expand, the loss of diversity could be arrested [e.g., 29].

## Conclusion

This work builds upon previous studies of the evolutionary relationships between western and eastern gorilla species [17], between *Pan *species [11], and among chimpanzee subspecies [12] in showing the prevalence of long-term post-divergence gene flow in African ape population histories. It is further apparent that African ape populations were subject to changes in size in the past, with evidence for both size decreases and expansions, and these are likely linked to changes in habitat caused by climate oscillations during the Pleistocene. It is plausible that habitat changes might also facilitate secondary introgression of already divergent populations, and human mediated habitat fragmentation has been invoked to explain numerous cases of secondary admixture in old world monkeys [19]. However, we find no support in our data for the postulated recent occurrence of secondary admixture of western lowland and Cross River gorillas [18]. Instead, we find that gene flow accompanied the divergence of western lowland and Cross River gorillas until just 400 or so years ago, which rather supports a scenario in which intensifying human activities may have increased the isolation of ape populations. The lack of a signal of secondary admixture in African apes is in notable contrast to the recent finding of substantive levels of introgression between the extinct Neanderthals and modern humans who were ancestral to populations found outside of Africa [20].

Coalescence-based approaches to inferring population histories using genetic data can reveal complex patterns of isolation with persistent migration or secondary contact that are difficult to reconcile with a view of evolutionary relationships as bifurcating processes that can be represented in a tree-like form. These results, along with earlier work on the patterns of morphological and phylogenetic relationships among baboon taxa, emphasize the challenges inherent to applying subspecies and species designations to dynamic entities like populations of organisms [7,52].

## Methods

### Sampling, DNA extraction and genotyping

Thirty-five gorilla specimens from the Cross River region on the border of Cameroon and Nigeria were obtained from the Museum of Natural History in Berlin, Germany. Twenty-six of the samples came from Ossidinge, a former missionary station located on the Cross River in Cameroon (5°15' - 6°15' N and 8°50' - 9°50' E; [53]). Although collected on expeditions in 1904-1907, some of these skulls were apparently obtained from local people and thus might have been older but not substantially so. The collection was transferred to Berlin in the early 1900s and stored at the Museum of Natural History since then.

Approximately 150 mg segments of tooth roots were extracted in physically-isolated laboratories dedicated to ancient DNA work following the protocol suggested in [54]. We first assessed whether we had recovered amplifiable nuclear DNA by attempting to amplify three autosomal microsatellites as well as a segment of the amelogenin gene in separate standard PCR reactions (see SI). The 14 samples for which at least two loci successfully amplified were then processed in a two-step multiplex approach [55] using the three original and five additional microsatellites (see SI).

Genotypes from 71 contemporary gorillas were generated using non-invasively collected fecal samples. The data from the Cross River population came from a previously published dataset [24]. For the demographic simulations we used genotypes from 92 western gorillas (*Gorilla gorilla gorilla*) (11 Cameroonian described in [17] and 81 from Gabon [56]). We included individuals from several locations in order to recover the genetic variation present in extant western gorillas. The data used from published work [17,24,56] employed noninvasive fecal samples from contemporary gorillas, collected in accordance with all relevant governmental guidelines and so further permissions were not necessary for the study presented here.

### Data analysis

We previously estimated the amount of repetition necessary to produce genotypes significantly likely to be free of errors due to allelic dropout or spurious alleles [55]. An individual genotype was considered to be confirmed when a homozygous allele was detected in at least six independent multiplex PCR replicates or when each of two apparently heterozygous alleles was observed in at least two multiplex PCR replicates [55]. Summary statistics were calculated as outlined in the Supplementary Information. We furthermore investigated whether or not individual loci in both the historical and the modern Cross River gorilla dataset show evidence for null alleles, small allele dominance (allelic dropout) or mis-scoring due to stuttering by using the program ***MICROCHECKER v2.2.3 ***[57].

We applied a randomization strategy in order to assess whether there was a significant difference in levels of genetic variation in the historical samples relative to the modern ones. We randomly drew a sample equal in size to the historical gorilla sample from the modern gorilla genotypes, and repeated this 10,000 times and computed the average expected heterozygosity (H_e_) over all loci which provides an unbiased estimate. We evaluated the significance level by estimating the percentage of resampling steps that provided a higher or equal H_e _than observed in the historical Cross River gorilla population.

An alternative approach of assessing levels of genetic variation while accounting for different sample sizes [58] is provided by allelic richness measurements as calculated with ***FSTAT v.2.9.3.2***. We also used this program to calculate F_IS _values [59] in order to estimate the probability of allelic identity due to population substructure or potential inbreeding.

### Effective population size

To generate short-term estimates of the effective population size (N_e_), we assumed that Cross River gorillas constitute an isolated population during the time spanned by our sampling (100 years). In general all models follow the same basic assumptions: the focal population is expected to be in panmixia, discrete generations exist and mutation, selection and migration are negligible compared to genetic drift, thus representing a simplified Wright-Fisher population [30,31]. Short-term N_e _estimators are based on the variance of allele frequency changes over time and hence provide a harmonic mean of N_e _for the time elapsed between the two sampling periods (for comprehensive reviews see [60]).

We calculated two moment estimators, one using the program ***MNE 1.0 ***[61,62] and applying Nei and Tajima's sampling scheme 1 [62]. The authors suggested two exclusive sampling schemes: plan 1 assumes that gene sampling happened after reproduction from a population in which census size approximates the effective size; plan 2 instead is based on the assumption that the sampled individuals do not contribute to the next generation and the census size is much larger than N_e_. The second moment estimator was calculated according to Waples' formula [63] also adopting Nei and Tajima's sampling plan 1. As simulations have shown that moment estimators may suffer from imprecision and overestimation of N_e _(e.g. [35]) whenever genetic drift is strong and genetic markers with high diversities are used, we accompanied these two moment estimators with three probabilistic ones. We used a pseudo-likelihood approach [61] as implemented in ***MNE 1.0 ***that also applies Nei and Tajima's sampling plan 1 but reduces multiallelic loci into a biallelic state by choosing one and pooling all other alleles, respectively. A second approach that utilizes the full allele spectra for multiallelic markers is based on a Monte Carlo evaluation to compute the likelihood of N_e _given the data and adopting sampling plan 2 according to Nei and Tajima (***CoNe***; [34,64]). Finally, we used a Bayesian approach coupled with Markov Chain Monte Carlo simulations that is based on the approximation of the gene genealogy by the coalescence principle and not the population gene frequency as in [64] (***tm3.1***; [34]). The authors implemented sampling plan 2 into their model.

When using these probabilistic approaches we commonly applied a prior N_e_-max of 3,000 and minimum run time of one million steps.

### Demographic inferences

We first assumed an evolutionary model in which the Cross River population diverged from the western gorilla population T_divergence _generations ago (Figure 2A). The two populations exchanged N_m _migrants per generation until T_migration _generations ago with T_migration _≤ T_divergence_. The effective population sizes of the ancestral gorilla population (N_ancestral_) and western lowland gorillas (N_western_) after the divergence of the Cross River population are assumed to be constant but are allowed to be different. The effective size of the Cross River population N_crossriver_old _is also assumed to be constant until T_bottleneck _generations when an exponential decrease began lasting until today and finally reducing the effective population size to N_crossriver_now_. All microsatellite loci were simulated assuming a stepwise mutation model with mutation rate μ with a prior distribution N (0.0004, 0.0001) truncated at 0.0002 and 0.0006 [12,65]. All prior distributions used for this model are given in Table 2 and the effective sizes are based on existing knowledge on the census size of the current Cross River population (< 300 individuals) or published estimates for western gorillas [13,17].

We also considered an alternative scenario, wherein the Cross River and the western gorillas did not exchange any migrants after their initial divergence. However, at a given time in the past (T_admixture_) a fraction of the Cross River population (γ_admixture_) was replaced by immigrants from the western gorillas (Figure 2B) and thus mirroring a hybridization scenario. For parameters common to both models we used the same prior distributions (Table 2). The prior distributions of T_admixture_, T_migration _and the prior distribution on (γ_admixture_) were chosen uniform within (0,1).

We used the software ***Serial SimCoal ***[66], a modified version of the program ***SimCoal ***[67], specifically designed to simulate temporally-spaced samples using a coalescent framework. The simulated data were then implemented in an Approximate Bayesian Computations framework (ABC) [68] in order to estimate parameters of the above model. We extended a methodology recently introduced and described in detail [69] to take the independence of the *K *studied loci explicitly into account (SI). This allowed us to reduce the necessary computations to a fraction of 1/K since we simulated a single locus rather than the complete set of observed loci, as was done previously. We successfully validated our approach (SI) using the methodology introduced in [39]. However, due to the low number of loci we report slight biases for some of our estimates: overestimation of N_crossriver_now_, underestimation of T_divergence _and a more global overestimation of N_ancestral _over the entire parameter space. Model comparison was performed using Bayes factors as described in [69], but adapted to the case of independent loci (SI).

Our inference relied upon the following summary statistics computed on the observed and simulated microsatellite loci: the variance in repeat length within each population and the difference in mean repeat length between all pairs of populations. We assumed a recently refined gorilla generation time of 20 years (IUCN Red List of Threatened Species™) and thus the historical samples were collected five generations before present.

## Authors' contributions

OT, DW and LV designed research; OT, DW, MS, CL performed research; OT, DW, MA, KG, analyzed data; LV provided materials; OT, DW, MA, RAB and LV wrote the paper. All authors read and approved the final manuscript.

## Supplementary Material

Additional file 1 **Supplementary Information**. Here we provide additional information regarding laboratory methods and data analyses. Click here for file
