# Supplementary material for: Historical sampling reveals dramatic demographic changes in western gorilla populations
Source: BMC Evol Biol. 2011 Apr 1;11:85. doi: 10.1186/1471-2148-11-85 (PMC3078889; doi:10.1186/1471-2148-11-85)
Supplement: Additional file 1 — Supplementary Information. Here we provide additional information regarding laboratory methods and data analyses. [file 1471-2148-11-85-S1.PDF]

Supplementary Information

Historical sampling reveals dramatic demographic changes in western gorilla populations

O. Thalmann, D. Wegmann, M. Spitzner, M. Arandjelovic, K. Guschanski, C. Leuenberger, R.A. Bergl and L. Vigilant

1 Laboratory Analysis

1.1 Initial PCR test and multiplex amplification

In order to assess whether or not the DNA extracted from 35 historical gorilla samples would allow amplification of autosomal microsatellite loci, we ran an initial PCR test on all extracted samples. Test amplifications of three autosomal microsatellites and one segment of the sex-specific amelogenin gene were set up in a laboratory dedicated for work on ancient materials and performed in a standard one step PCR of 20 μl volume consisting of 1x Super Taq PCR buffer (HT Biotechnology) already containing $MgCl_2$, an additional 0.88 mM $MgCl_2$, 0.2 μM each forward and reverse primer, 0.125 mM each dNTP, 0.5 U Super Taq (HT Biotechnology) pre-mixed 2:1 with 1 μg/μl TaqStart monoclonal antibody (BD Biosciences) to facilitate hot start conditions and 5μl of template DNA. PCR amplifications on a Peltier cycler PTC 200 (MJ Research) included the following steps: initial denaturation for 9 min at 94°C, 50 cycles each of 20 sec at 94°C, 30 sec at X°C (dependent on the primers used), 30 sec at 72°C, and a final elongation for 30 min at 72°C. PCR products were visualized under UV light using a 2.5% agarose gel containing ethidium bromide. Extracts from twenty-one specimens produced no or only sporadic amplifications, and so we continued work with the 14 extracts which amplified products of the expected size at two or more loci.

The 14 extracts were subsequently subjected to a multiplex PCR approach, which requires two steps; one multiplex-step in which all non-labeled primers are pooled for an initial amplification and a second step using an aliquot of 1:20 diluted multiplex PCR product as a template for independent singleplex PCRs with each dye-labeled primer pair. The exact protocol used is detailed in Arandjelovic and colleagues, which also reports PCR success rates, allelic dropout incidence, and the frequency of false alleles experienced using these extracts (Arandjelovic *et al.*, 2009). Three replicates of 5 μl from each extract, as well as the extraction blanks included into each respective extraction series and at least three negative controls (5 μl ddH2O in place of the DNA), were included in each 20 μl PCR set up. Each PCR product amplified in the two-step multiplex approach was visualized under UV light using a 2.5% agarose gel containing ethidium bromide and prepared for further analysis on an ABI 3700 Genetic Analyzer (Applied Biosystems) according to the manufacturer's instructions. PCR products labeled with different dyes were combined and run against an internal size standard (ROX labeled HD400). Alleles were called using `GeneMapper v. 3.7` (Applied Biosystems) and independently confirmed by two researchers (OT and KG). As a precautionary measure, we genotyped all researchers conducting laboratory work (OT and MS) and limited the handling of the historical extracts to only a single researcher (OT).

Historical DNA, particularly from close human relatives such as African apes, is susceptible to contamination by modern DNA. None of our extraction blanks yielded any positive amplification with the eight microsatellite loci. Of 168 negative controls performed (7 multiplex PCR $\times$ 3 replicates $\times$ 8 loci) we only observed nine sporadic amplifications (5.36%). In those cases we repeated the singleplex

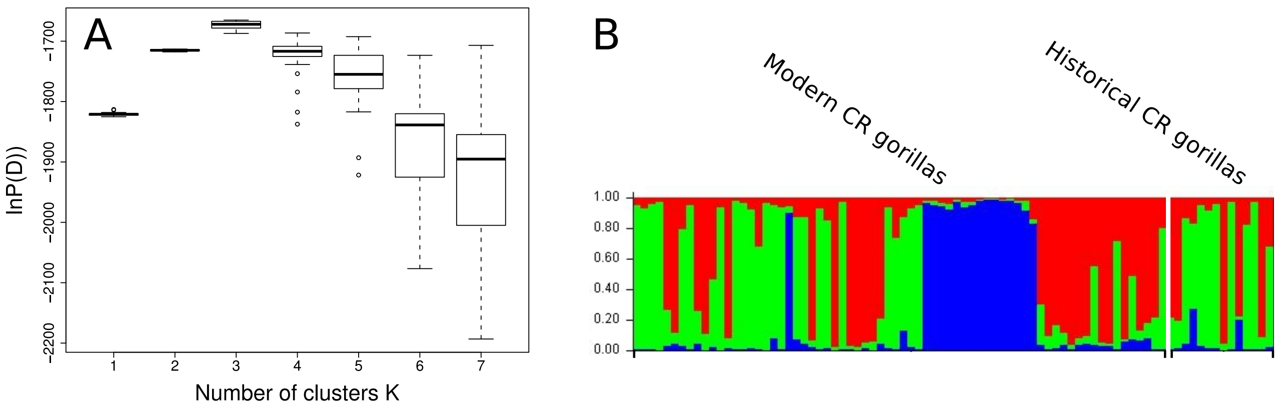

Figure 1. Structure Analysis
A) Likelihood distribution of different K.
B) Admixture probabilities for each Cross River gorilla used in this study, modern and historical samples respectively. Modern CR gorillas and Historical CR gorillas are divided here with a solid white line. Each colored bar represents the proportion of ancestry for each single individual under the assumption of K = 3 populations.

reaction for the respective locus. Only once did we need to repeat the multiplex reaction due to the occurrence of consistent amplification products in the same negative control in two singleplex reactions using one locus (VWF).

Because the concentration of amplifiable DNA in the extracts was so low (< 1 pg/μl, (Arandjelovic *et al.*, 2009)), there was a higher chance of 'allelic dropout', the stochastic nonamplification of one allele at a heterozygous locus. Thus, all heterozygous loci were confirmed by detection of each allele at least two times in independent PCRs, and each apparently homozygous locus was accepted after the allele was observed in six independent PCRs, as previously described (Arandjelovic *et al.*, 2009).

One locus (D5S1470) showed an unusual allele range, and resequencing of a subset of individuals from the modern Cross River population revealed a two basepair insertion/deletion polymorphism in the region adjacent to the microsatellite. Given the complex nature of this microsatellite and the dependency of simulations upon estimated mutation rates, we excluded this locus when performing demographic simulations.

1.2 Data Analysis

Using `Arlequin v.3.1` (Excoffier *et al.*, 2005) we calculated the number of alleles, allele frequencies and observed/expected heterozygosities (H_o/H_e) for each locus. We tested each locus for deviations from Hardy-Weinberg equilibrium (HWE) and pairs of loci for deviations from linkage disequilibrium. We adjusted the significance threshold for non-independent tests according to Cross and Chaffin (Cross and Chaffin, 1982). An additional test for deviation from HWE was performed using the excess and deficiency of heterozygotes as well as probability tests at each locus and then applying a global test over all loci as implemented in `Genepop v.4` (Rousset, 2008). Population differentiation tests were also performed with the aforementioned software. All tests in `Genepop v.4` using Markov chains were run with the following parameters: 100,000 dememorizations, 1,000 batches and 10,000 iterations per batch.

In order to observe any potential outlier in our historical dataset, we performed multiple `Structure` (Pritchard *et al.*, 2000) analyses under the following conditions: 100,000 burn in steps followed by 100,000 steps from which the parameter were recorded. We assumed an admixture model and found that the most likely K value was 3 (as estimated from lnP(D)). The results also demonstrate that the admixture pattern of historical Cross River samples does not differ from that of the modern samples, indicating that the historical samples constitute a subset of contemporary Cross River gorillas (Figure 1).

2 Demographic Modeling

2.1 Approximate Bayesian Computation

Parameter inference and model selection was performed using an Approximate Bayesian Computations (ABC) approach taking the independence of the studied loci explicitly into account. Consider genetic data at a single locus generated under a model $\mathcal{M}$, determined by the parameters $\boldsymbol{\theta}$, whose joint prior density is denoted by $\pi(\boldsymbol{\theta})$. The genetic data is further replaced by a set of summary statistics $\mathbf{s}$, and the quantity of interest is the posterior distribution

$$\pi(\boldsymbol{\theta}|\mathbf{s_{obs}}) \propto \pi(\boldsymbol{\theta}) f_{\mathcal{M}}(\mathbf{s_{obs}}), \tag{1}$$

where $f_{\mathcal{M}}(\mathbf{s_{obs}})$ is the likelihood of the observed summary statistics $\mathbf{s_{obs}}$. Since the likelihood function of the considered models cannot be calculated analytically we use stochastic simulations: we simulate for a parameter vector $\boldsymbol{\theta}$, drawn from the prior $\pi(\boldsymbol{\theta})$, the summary statistics $\mathbf{s}$ from the model $\mathcal{M}$ and retain only the parameter vector if $dist(\mathbf{s}, \mathbf{s_{obs}}) < \epsilon$, *i.e.* if the simulated summary statistics $\mathbf{s}$ are sufficiently close to the observed summary statistics $\mathbf{s_{obs}}$. Let us denote the likelihood of the truncated model $\mathcal{M}_\epsilon(\mathbf{s}_{obs})$ obtained by this acceptance-rejection process as $f_\epsilon(\mathbf{s}|\boldsymbol{\theta})$ and the truncated prior (the distribution of the parameters retained after the rejection process) by $\pi_\epsilon(\boldsymbol{\theta})$. Recently, Leuenberger and Wegmann (2010) have shown that the posterior distribution of the parameters under the full model $\mathcal{M}$ is exactly equal to the posterior distribution under the truncated model $\mathcal{M}_\epsilon(\mathbf{s}_{obs})$ given by

$$\pi(\boldsymbol{\theta}|\mathbf{s}_{obs}) \propto \pi_\epsilon(\boldsymbol{\theta}) f_\epsilon(\mathbf{s}_{obs}|\boldsymbol{\theta})\pi_\epsilon(\boldsymbol{\theta}). \tag{2}$$

Given the observed summary statistics $\mathbf{s}_{obs} = (\mathbf{s}^1_{obs}, \ldots, \mathbf{s}^K_{obs})$ at K independent loci, the posterior distribution is given by

$$\pi(\boldsymbol{\theta}|\mathbf{s}_{obs}) \propto \pi_\epsilon(\boldsymbol{\theta}) \prod_{k=1}^{K} f_\epsilon(\mathbf{s}^k_{obs}|\boldsymbol{\theta}), \tag{3}$$

where simulations of a single locus are accepted if

$$\max_{1\le k\le K} dist(\mathbf{s}, \mathbf{s}^k_{obs}) < \delta_{crit} \tag{4}$$

and rejected otherwise. The threshold δ_{crit} is a distance chosen such that an arbitrary fraction of all simulations meet the criteria. Following Leuenberger and Wegmann (2010) we assume the truncated model to be a general linear model, we thus assume the summary statistics $\mathbf{s}$ created by the truncated model's likelihood $f_\epsilon(\mathbf{s}|\boldsymbol{\theta})$ to satisfy

$$\mathbf{s}|\boldsymbol{\theta} = \mathbf{C}\boldsymbol{\theta} + \mathbf{c}_0 + \boldsymbol{\epsilon}, \tag{5}$$

where $\mathbf{C}$ is a matrix of constants, $\mathbf{c}_0$ a vector and $\boldsymbol{\epsilon}$ a random vector with a multivariate normal distribution of zero mean and covariance matrix $\boldsymbol{\Sigma}_s$:

$$\boldsymbol{\epsilon} \sim \mathcal{N}(\mathbf{0}, \boldsymbol{\Sigma}_s).$$

The matrices $\mathbf{C}$, $\boldsymbol{\Sigma}_s$ and the vector $\mathbf{c}_0$ are estimated from the retained simulations (see Leuenberger and Wegmann (2010) for details). Compared to previous ABC approaches (e.g. Wegmann and Excoffier (2010)) the methodology proposed here reduces the computations to a fraction $1/K$ since only a single locus is simulated, rather than a set of K loci. We implemented the proposed approach in the software package `ABCtoolbox` (Wegmann *et al.*, 2010), an updated version of which will be available at *www.popgen.unibe.ch.*

2.2 Model Selection

In order to perform model comparisons using Bayes factors we estimated the marginal densities of model $\mathcal{M}$ at $\mathbf{s_{obs}}$. Under the above assumptions and denoting the acceptance rate of the rejection

process by $A_\epsilon(\mathbf{s}_{obs}, \pi) = N/M$, where N is the number of retained values and M the total number of simulations performed, LEUENBERGER and WEGMANN (2010) estimated the marginal density for a single locus as follows:

$$f_{\mathcal{M}}(\mathbf{s}_{obs}) = \frac{A_\epsilon(\mathbf{s}_{obs}, \pi)}{N|2\pi\mathbf{D}|^{1/2}} \cdot \sum_{j=1}^{N} e^{-\frac{1}{2}(\mathbf{s}_{obs}-\mathbf{m}^j)^t\mathbf{D}^{-1}(\mathbf{s}_{obs}-\mathbf{m}^j)} \tag{6}$$

where the sum runs over the parameter sample $\mathcal{P} = \{\boldsymbol{\theta}^1, \ldots, \boldsymbol{\theta}^N\}$,

$$\mathbf{D} = \mathbf{\Sigma}_s + \mathbf{C}\mathbf{\Sigma}_\theta\mathbf{C}^t$$

and

$$\mathbf{m}^j = \mathbf{c}_0 + \mathbf{C}\boldsymbol{\theta}^j.$$

$\mathbf{\Sigma}_\theta$ is the matrix that has been chosen for the widths of the Gaussian peaks to smooth the distribution of retained parameters (see LEUENBERGER and WEGMANN (2010)).
For K independent loci the marginal density is given by

$$f_{\mathcal{M}}(\mathbf{s}_{obs}) = \frac{A_\epsilon(\mathbf{s}_{obs}, \pi)}{N\prod_{k=1}^{K}|2\pi\mathbf{D}|^{1/2}} \cdot \sum_{j=1}^{N}\prod_{k=1}^{K} e^{-\frac{1}{2}(\mathbf{s}_{obs}-\mathbf{m}^j)^t\mathbf{D}^{-1}(\mathbf{s}_{obs}-\mathbf{m}^j)} \tag{7}$$

For two models $\mathcal{M}_A$ and $\mathcal{M}_B$ with prior probabilities π_A and $\pi_B = 1 - \pi_A$, the Bayes factor B_{AB} in favor of model $\mathcal{M}_A$ over model $\mathcal{M}_B$ is

$$B_{AB} = \frac{f_{\mathcal{M}_A}(\mathbf{s}_{obs})}{f_{\mathcal{M}_B}(\mathbf{s}_{obs})} \tag{8}$$

where the marginal densities $f_{\mathcal{M}_A}$ and $f_{\mathcal{M}_B}$ are calculated according to (7).

2.3 Variation in parameter estimates

We decided to base the parameter estimates and model selection on the 5,000 simulations closest to the observed data sets. To check the robustness of our choice we repeated the ABC estimation with different numbers of retained simulations, ranging from 2,500 to 20,000. These results are shown in Figure 2. This analysis suggests that our estimates are very robust to changes in the number of simulations accepted in the rejection step.

2.4 Validation of parameter inference

Unbiased posterior distributions have a well-balanced coverage property, such that the true value of a parameter should be found q percent of the time in a q% credible interval (WEGMANN *et al.*, 2009). We investigated this behavior for our preferred model by creating 2,500 artificial data sets matching the observed data set in number of loci for each model, randomly drawing each time parameters from our prior distribution. We then estimated the parameter posterior distributions as described above and estimated the proportion of the true parameters contained in 10%, 25%, 50%, 75% and 90% credible intervals. Following PETER *et al.* (2010), we examined these coverage properties locally for different parameter values. We thus divided our 2,500 simulated data sets into 15 equally sized parameter bins according to the value of the estimated posterior mode, for each parameter independently. The results of this analysis is given in Figure 3. Towards the borders of the priors we observed very few modes, partly due to smoothing. This leads to inaccurate estimation of the coverage property (especially with non-uniform priors) or, in extreme cases, no estimates at all. While it proved difficult to obtain unbiased estimates over the entire parameter range, the accuracy for posteriors similar to those obtained for the Cross River data set (indicated by the red lines in Figure 3) was encouraging.

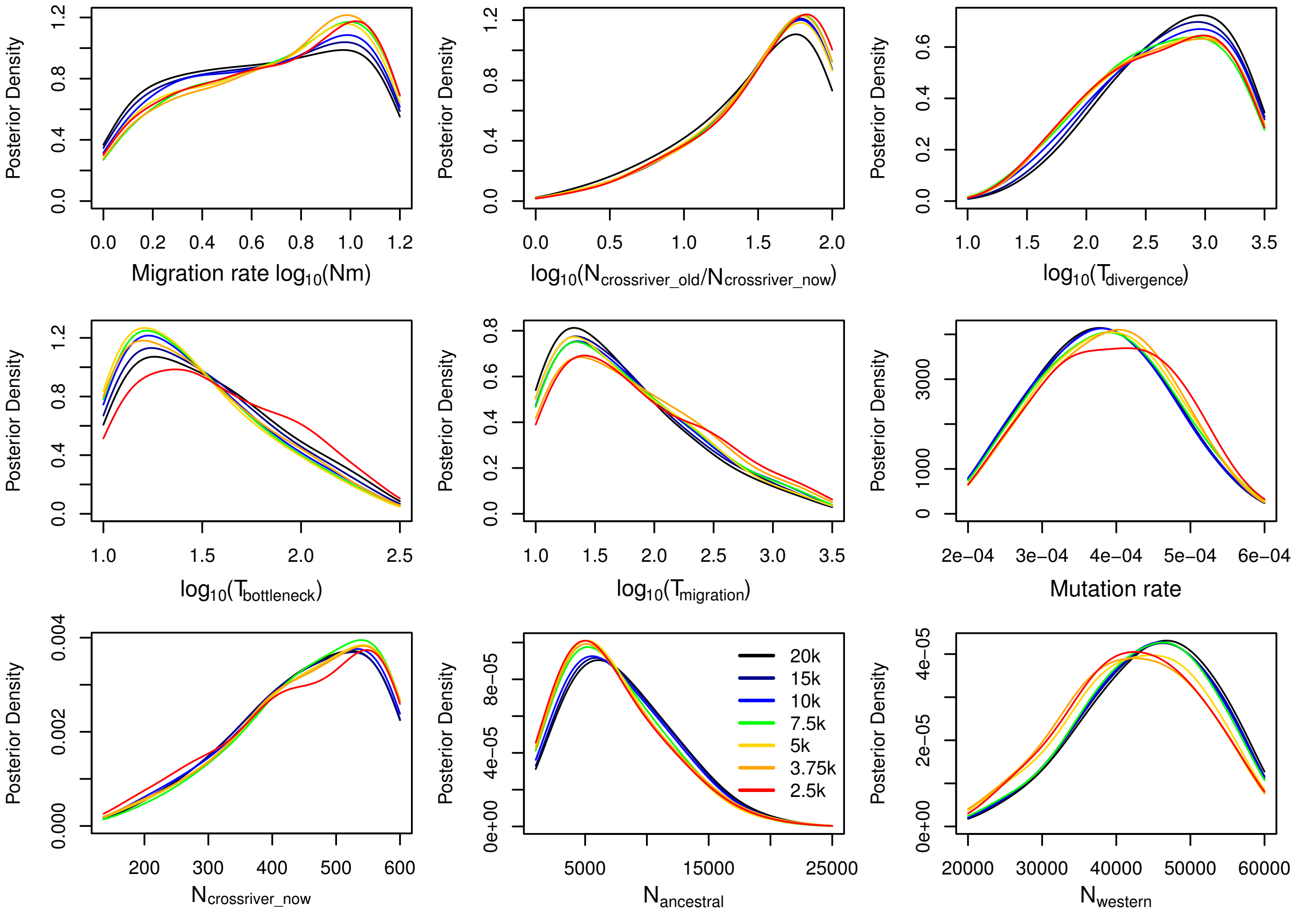

Figure 2. Posterior estimate robustness
For each parameter, the colored curves give its posterior distribution with different numbers of simulations retained in the rejection step. Note that the values for effective population size (N) estimates are given in gene copies so they are twice the number of individuals.

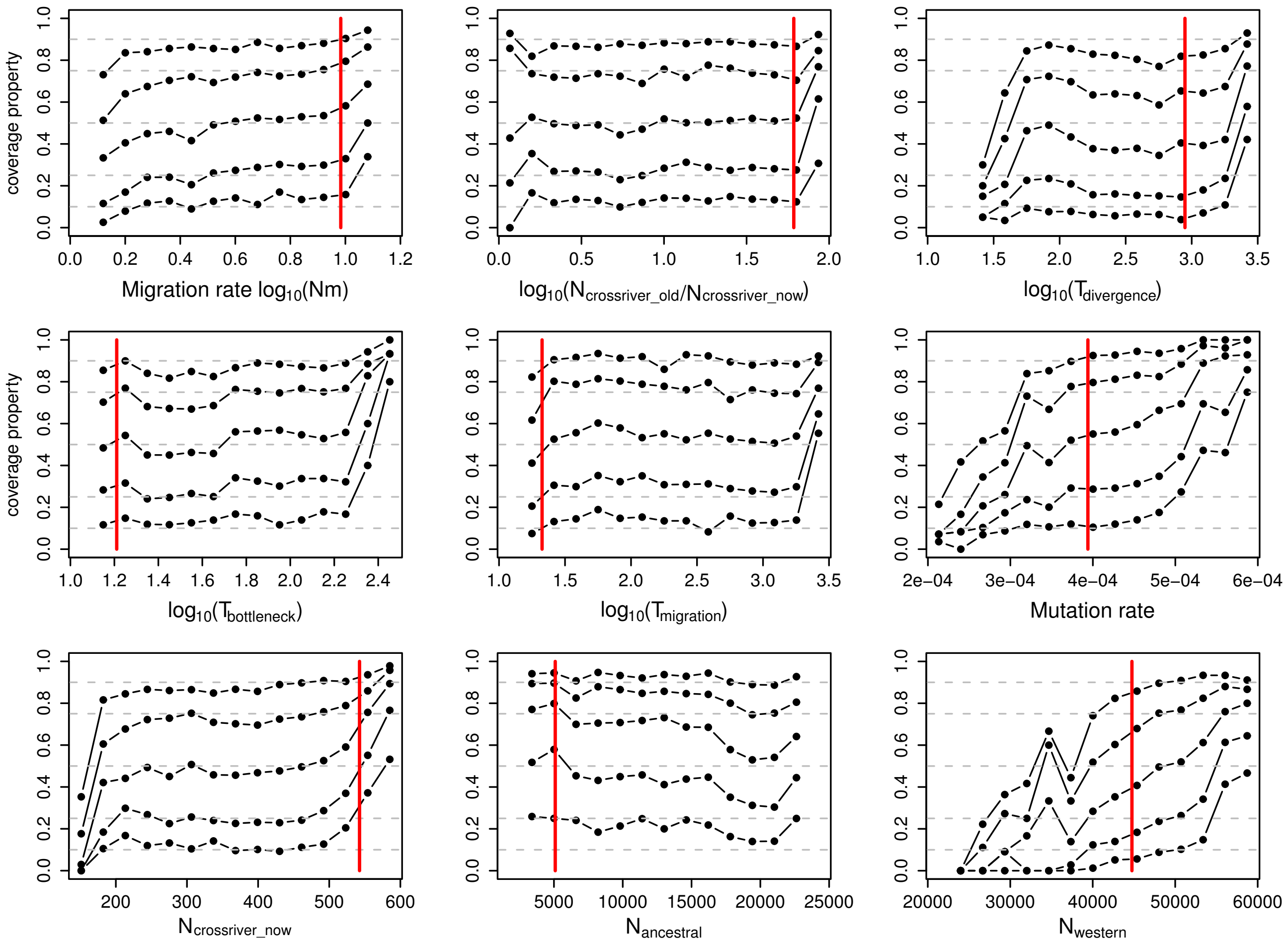

Figure 3. Local coverage properties for the isolation with migration model
We simulated 2,500 data sets with parameter values drawn from the prior distribution. We then grouped all obtained posterior distributions into 15 equally sized bins according to the mode of that distribution (see text for further details). We defined the credible intervals as follows 10%, 25%, 50%, 75% and 90% and show these here as grey dashed lines. The coverage property is finally computed as the proportion of the true parameters contained in those credible intervals (black dots). The mode of the gorilla data set is given by red vertical line. Note that the values for effective population size (N) estimates are given in gene copies so they are twice the number of individuals.

References

Arandjelovic, M., K. Guschanski, G. Schubert, T. Harris, O. Thalmann, *et al.*, 2009 Two-step multiplex polymerase chain reaction improves the speed and accuracy of genotyping using DNA from noninvasive and museum samples. Molecular Ecology Resources **9**: 28–36.

Cross, E. M., and W. W. Chaffin, 1982 Use of the binomial theorem in interpreting results of multiple tests of significance. Educational and Psychological Measurement **42**: 25–34.

Excoffier, L., G. Laval, and S. Schneider, 2005 Arlequin (version 3.0): An integrated software package for population genetics data analysis. Evolutionary Bioinformatics Online **1**: 47–50.

Leuenberger, C., and D. Wegmann, 2010 Bayesian computation and model selection without likelihoods. Genetics **184**: 243–252.

Peter, B., D. Wegmann, and L. Excoffier, 2010 Distinguishing between population bottleneck and population subdivision by a Bayesian model choice procedure. Molecular Ecology **19**: 4648–4660.

Pritchard, J., M. Stephens, and P. Donnelly, 2000 Inference of population structure using multilocus genotype data. Genetics **155**: 945–959.

Rousset, F., 2008 genepop007: a complete re-implementation of the genepop software for Windows and Linux. Molecular Ecology Resources **8**: 103–106.

Wegmann, D., and L. Excoffier, 2010 Bayesian inference of the demographic history of chimpanzees. Mol Biol Evol **27**: 1425–1435.

Wegmann, D., C. Leuenberger, and L. Excoffier, 2009 Efficient Approximate Bayesian Computation coupled with Markov Chain Monte Carlo without likelihood. Genetics **182**: 1207–1218.

Wegmann, D., C. Leuenberger, S. Neuenschwander, and L. Excoffier, 2010 ABCtoolbox: a versatile toolkit for Approximate Bayesian Computations. BMC Bioinformatics **11**: 116.

3 Supplementary Tables

Table S1: List of genotypes determined for historical Cross River gorilla. (— indicates missing data)

Sample	D1S550	D5S1457	D5S1470	D7S817	D8S1106	D10S1432	D16S2624	VWF
406	188/184	116/116	172/162	134/134	141/141	135/135	142/142	157/153
806	188/188	128/120	166/162	138/114	145/141	155/139	142/138	149/145
1206	188/172	132/124	164/160	142/134	141/141	151/151	142/138	161/157
1306	—/—	136/120	—/158	134/114	141/137	151/135	142/138	145/145
1506	184/172	120/116	176/168	134/114	145/141	151/135	142/142	149/149
1806	188/184	124/116	160/158	142/134	141/141	135/131	142/138	149/145
2006	184/172	124/120	174/164	142/138	141/141	135/135	142/142	149/145
10	188/188	120/116	162/162	134/130	141/141	155/139	142/142	157/145
48	188/184	128/116	178/162	138/114	145/141	147/147	142/138	149/149
52	192/188	132/116	176/162	130/114	141/137	155/135	142/138	153/153
53	188/188	120/116	166/158	134/114	145/145	151/147	142/142	157/145
62	188/184	128/116	178/162	138/114	145/141	147/147	142/138	149/149
65	192/188	120/116	168/—	134/134	145/141	—/139	146/142	153/149
68	—/184	124/116	174/162	142/114	141/141	139/135	142/138	149/145

Table S2: Standard summary statistics

Locus	Historical Gorillas				Contemporary Gorillas			
	#gene copies	#alleles	H_o[a]	H_e[b]	#gene copies	#alleles	H_o[a]	H_e[b]
D1S550	25	4	0.75	0.657	135	8	0.687	0.712
D5S1457	28	6	0.929	0.772	136	6	0.706	0.71
D5S1470	26	10	0.917	0.883	138	10	0.739	0.825
D7S817	28	5	0.857	0.772	136	5	0.731	0.695
D8S1106	28	3	0.5	0.489	136	4	0.397	0.559
D10S1432	27	6	0.615	0.815	136	8	0.806	0.764
D16S2624	28	3	0.643	0.474	133	3	0.492	0.533
VWF	28	5	0.643	0.749	135	5	0.493	0.637
Mean	27.25	5.25	0.732	0.701	135.63	6.13	0.631	0.679

[a]observed heterozygosity
[b]expected heterozygosity

Table S3: Fixation index and Allelic richness

Locus	Historical Gorillas		Contemporary Gorillas	
	FIS	Allelic richness	FIS	Allelic richness
D1S550	-0.165	4.000	0.030	4.915
D5S1457	-0.211	5.840	0.006	4.965
D5S1470	-0.052	10.000	0.105*	7.134
D7S817	-0.114	4.984	-0.050	4.028
D8S1106	-0.022	2.984	0.291*	3.293
D10S1432	0.247	5.923	-0.048	6.321
D16S2624	-0.376	2.857	0.077	2.563
VWF	0.146	4.857	0.228*	3.838
mean	-0.050		0.072*	

* fewer than 5% of the randomizations gave higher FIS values than observed
